# Autoimmune thyroid status in subclinical thyroid disorders in patients attending a tertiary care center in Nepal: a hospital-based cross-sectional study

**DOI:** 10.1186/s12902-023-01480-6

**Published:** 2023-10-11

**Authors:** Vijay Kumar Sharma, Apeksha Niraula, Eans Tara Tuladhar, Aseem Bhattarai, Mithileshwer Raut, Raju Kumar Dubey, Sujata Baidya, Naresh Parajuli

**Affiliations:** 1 Department of Clinical Biochemistry, Maharajgunj Medical Campus, Institute of Medicine, Tribhuvan University Teaching Hospital, Kathmandu, Nepal; 2 Department of Internal Medicine (Endocrinology), Maharajgunj Medical Campus, Institute of Medicine, Tribhuvan University Teaching Hospital, Kathmandu, Nepal

**Keywords:** Subclinical hyperthyroidism, Subclinical hypothyroidism, Anti-thyroid peroxidase, Anti-thyroglobulin, Anti-thyroid stimulating hormone receptor, Hashimoto’s thyroiditis, Grave’s disease

## Abstract

**Background:**

Thyroid dysfunction is the leading endocrine disorder worldwide. Iodine deficiency disorders, which were once the major etiology of thyroid dysfunctions, now have been succeeded by autoimmune thyroid diseases with the rise in aberrant salt ionization protocols. This study endeavors to access the level of thyroid autoantibodies viz. anti-thyroid peroxidase (anti-TPO), anti-thyroglobulin (TGA), and anti-thyroid stimulating hormone receptor (TRAb) in individuals with subnormal thyroid profiles.

**Methods:**

This hospital-based cross-sectional study was conducted at the Department of Clinical Biochemistry, Tribhuvan University for a period of six months. Using non-probability (purposive) sampling method, a total of 60 patients were enrolled with subnormal thyroid profiles to include the population who have not yet started medication. Thyroid hormones (free T3, free T4, TSH) and thyroid antibodies (anti-TPO, TGA, and TRAb) were measured. For non-parametric data, Chi-square test and Kruskal-Wallis test were used. Spearman’s correlation was done to determine the association between variables.

**Results:**

Out of 60 participants, the majority of the population between 25 and 44 years were diagnosed with thyroid dysfunction with female preponderance. Among all, 40% (n = 24) had subclinical hyperthyroid states while, 60% (n = 36) had subclinical hypothyroid states, and 75% (n = 45) of the total exhibited positive thyroid antibodies. In subclinical hypothyroid patients with TSH above 10 µIU/ml, anti TPO (58.5%) and TGA (66.7%) positivity were highly prevalent. On the other hand, TRAb was exclusively positive in hyperthyroid condition (50% among the group) which is by far the first of its kind reported in Nepal.

**Conclusion:**

The rise in autoimmune thyroid disease among the Nepalese population infers that addressing iodine deficiency simply through salt iodinization may not be adequate to deal with the rising burden of thyroid disorders, especially in iodine-depleted areas. Also, the increasing prevalence of thyroid autoantibodies positivity in subclinical hypothyroidism in the Nepalese population accounts for the arduous screening and monitoring of autoimmune thyroid disorders in Nepal.

## Background

Thyroid gland abnormalities have afflicted a high number of populations, globally [1]. Precise diagnosis mandates accurate testing for free triiodothyronine (fT3), free thyroxine (fT4) and thyroid-stimulating hormone (TSH) commonly known as thyroid function tests. Any abnormality in thyroid function test warrants the clinicians to evaluate for thyroid antibodies titer [1, 2]. Hashimoto’s thyroiditis and Graves’ disease, two of the most prevalent autoimmune diseases that affect particular organs, are examples of autoimmune thyroid disorders [3, 4]. The pathogenesis of the illness has been linked to several predisposing genetic loci, including CTLA4, HLA, and IL2RA, as well as some environmental variables, including radioiodine therapy, iodine deficiency, and smoking [5, 6]. Although only 1% of people have autoimmune thyroid disease (AITDs), 15% of people with normal thyroid function may have subclinical and localized thyroiditis and circulating antithyroid antibodies [7]. Antibodies called anti-thyroid peroxidase (Anti-TPO) develop against a thyrocyte transmembrane protein that is implicated in the production of thyroid hormone. Thyroglobulin, a forerunner to thyroid hormone, is the target of anti-thyroglobulin (TG-Ab) antibodies [4]. Since these antibodies are found in more than 90% of cases of Hashimoto’s thyroiditis and more than 80% of cases of Graves’ disease, anti-TPO antibodies (previously known as anti-thyroid microsomal antibodies) and anti-TG antibodies are regarded as diagnostic of AITDs [4]. Anti-TPO and anti-TG antibodies are related to levels of thyroid-stimulating hormone (TSH) and both alone or in combination have been used to predict the development of hypo-/hyperthyroidism. As per the various research, the development of hypothyroidism in euthyroid subjects has been linked to altered levels of anti-thyroid antibodies and TSH [8, 9]. The etiology of Grave’s disease is primarily characterized by the autoimmune production of TSH receptor antibodies (TR-Ab). TR-Ab is heterogeneous and may either stimulate the TSH receptor (TS-Ab, TSH receptor stimulating antibody) or inhibit it (TB-Ab, TSH receptor blocking antibody), or it may have no impact at all. The predominant type in Graves’ Disease hyperthyroidism is TS-Ab [10, 11]. Abnormal serum thyrotropin (TSH) levels but normal serum-free thyroxine (fT4) and triiodothyronine (fT3) levels are indicative of subclinical thyroid disease [12, 13]. When TSH is 10.0 mIU/ml or higher, subclinical hypothyroidism is linked to an increased chance of cardiovascular events so early thyroid screening should be sought for timely intervention [14]. TSH levels above 10 mIU/ml signify severe thyroid dysfunction, while those between 5 and 10 mIU/ml allude to mild elevation [15]. There are a number of conditions that results in asymptomatic hypothyroidism, the most common being Hashimoto thyroiditis, followed by a history of thyroid surgery, the use of medications like lithium, anti-cancer medications, and amiodarone, etc. [15]. Factually, anti-thyroid antibodies are involved in the pathogenesis of autoimmune thyroiditis through complement-dependent cytotoxicity [16], thereby their appearance may actually predate the onset of overt thyroid disease or abnormal thyroid function tests over a number of years [17]. Follow-up thyroid profile testing in anti-thyroid antibody-positive people is crucial for prompt diagnosis because anti-thyroid antibodies have been found in healthy individuals, particularly females [3, 18]. Over time, there has been a steady rise in the use of biomarkers to assess thyroid autoimmune risk and prognosis. Thyroid autoantibodies and genetic susceptibility testing are extremely prognostic of thyroid autoimmunity and thyroid dysfunction in the future [19].

Surprisingly, studies carried out in various regions of Nepal reveal a significant prevalence of thyroid dysfunction, ranging from 22.42 to 36% [20–23]. Few types of studies have used Anti-TPO as a biomarker for identifying autoimmune thyroid disease in Nepal, which limits the amount of research that has been done on the autoimmune profile in thyroid disease. Anti-TPO positivity has been found in 26.7% of the group studied by researchers [24], and upto 3.26% in pregnant women [25]. Additionally, research studies among children revealed that 25.8% of patients had elevated TgAb (> 30 IU/ml), indicating thyroid autoimmune condition [26]. However, it is unknown how common anti-thyroid antibodies are in the general population. In addition, we do not have reported data attributing to the relationship between autoimmune profile and thyroid state (TSH, fT4, and fT3). Thus, identifying a group of individuals with abnormal thyroid profiles who also require thyroid autoantibody screening to rule out underlying autoimmune processes could be accomplished by examining the relationship between anti-thyroid antibodies and thyroid profile testing. The prevalence of subclinical hyperthyroidism is nearly 6% while that of subclinical hypothyroidism nearly 12% in the general population [27]. Researchers should evaluate the likelihood of the onset of overt diseases and their long-term health effects given the high prevalence of subclinical thyroid disorders in the general population [27].

Hence, this study intends to determine the status of anti-thyroid antibodies namely anti-thyroid peroxidase antibody, anti-thyroglobulin antibody, and thyroid stimulating hormone receptor antibody in patients with deranged thyroid profiles.

## Methods

This hospital-based observational (descriptive) study was conducted at the Department of Clinical Biochemistry in collaboration with the Department of Internal Medicine (endocrinology), TUTH for a period of 6 months. i.e., from 15th February, 2022 to 15th August, 2022. Nonprobability sampling (Purposive sampling) was used to include the patients who had not yet started medication. Samples of the patients processed in the clinical biochemistry laboratory with a deranged thyroid profile were incorporated after taking their clinical details. A total of sixty samples were included for the estimation of thyroid autoantibodies. All the samples that were processed in the clinical biochemistry laboratory, TUTH for thyroid function tests (fT3, fT4, and TSH) with any of the parameters having deranged values were included for antithyroid antibody estimation.

Values were considered deranged if any of the parameters i.e., fT3, fT4, and TSH were above or below the assay range.

- The assay ranges are given below:
  fT3 = 2.63–5.69 pmol/L.
  fT4 = 9.00- 19.04 pmol/L.
  TSH = 0.35 µIU/mL to 4.94 µIU/mL.
  Anti-TPO = < 30 IU/ml.
  TG-Ab = < 95.0 IU/ml.
  TR-Ab = < 1.5 IU/L.

We included the demographic variables like age, geographic location and ethnicity. Biochemical variables included were serum fT3, fT4 and TSH estimated by the chemiluminescence immunoassay technique in the Abbott ci4100 autoanalyzer. Anti-TPO, TR-Ab, and TG-Ab were estimated by chemiluminescence immunoassay in Maglumi 800 (Snibe Diagnostics) autoanalyzer.

Age period of participants was classified according to the criteria defined by World Health Organization (WHO) [28];

Children: 0–13 years.

Adolescent and crown of youth: 13–25 years.

Young adults:25–44 years.

Middle-aged adults: 44–60 years.

Elderly: 60–75 years.

Senile: 75–90 years.

Long-livers: >90 years.

A self-designed semi-structured proforma was used to record the data of the patients including the demographic and biochemical measurements. Sample handling and processing were done following the standard protocol under the aseptic technique.

### Statistics

Data was entered in MS Excel 2010 and analyzed with Statistical Package for Social Sciences (SPSS version 22.0). The normality of the data was checked using the Kolmogorov-Smirnov test. For descriptive statistics, mean, standard deviation, percentage and range were calculated. For parametric variables, students’ “t” test, and for non-parametric variables, Chi-square test and Kruskal-Wallis tests were used. Correlation between patient age, fT3, fT4, TSH and Anti-TPO, TR-Ab, and TG-Ab was determined using Pearson’s or Spearman’s correlation coefficient. p-value ≤ 0.05 was considered statistically significant.

## Results

### Demographic and socio-ethnic characteristics of the study population

A total of 60 samples were assessed whose TSH values were out of normal reference range. We analyzed the following variables: Age, Gender, Free T3, Free T4, TSH, Anti-TPO, TGA and TRAb. None of the variables were normally distributed. Among the total participants, females were preponderant (68.3%, n = 41) as compared to males (31.7%, n = 19). The participants ranged from minimum age of 18 years to a maximum of 79 years (Median-38.5; IQR-29,55.5) as depicted in Table 1 and Table 2. The thyroid dysfunction was exhibited more commonly in young adult age group below 45 years.

**Table 1:** Age and Gender-wise Distribution of Thyroid Status

Age Range	Clinical Status			
	Subclinical Hyperthyroid		Subclinical Hypothyroid	
	Male	Female	Male	Female
**Adolescent**	0	0	1	3
**Young adults**	4	8	3	13
**Middle-aged adults**	2	5	6	5
**Elderly**	1	3	2	3
**Senile**	0	1	0	0
**Total**	**7**	**17**	**12**	**24**

Among the recruited patients, 40% (n = 24) had subclinical hyperthyroid status while majority of the patients accounting for 60% (n = 36) had subclinical hypothyroid status. The gender-wise distribution depicted that both subclinical hyper- and hypothyroid status was preponderant in females, up to 70.83% (n = 17) and 66.67% (n = 24) respectively

**Table 2:** Descriptive statistics of study variables

Parameters	Subclinical Hyperthyroid (n = 24)	Subclinical Hypothyroid (n = 36)	p-value
Free T3	6.14 ± 1.82	3.45 ± 1.01	0.309
Free T4	19.72 ± 7.16	12.29 ± 1.65	0.287
TSH	0.028 ± 0.048	14.58 ± 10.92	0.097

### Autoimmune thyroid profile and clinical thyroid status

Among the study population, 75% (n = 45) unveiled positive thyroid autoantibodies (any of the three antibodies), among which 13.3% (n = 8) had only one antibody positive, 50% (n = 30) had two antibodies positive while 11.7% (n = 7) had all the three antibodies positive. The positive titre of thyroid antibodies was higher in females as compared to males; however, it was statistically insignificant. Majority of the females with positive thyroid autoantibodies were young adults (25–44 years) i.e. in the reproductive age group.

There was a statistically significant relation between subclinical thyroid status and positive rates of thyroid autoantibodies. A high positivity rate of thyroid autoantibody was seen in subclinical hypothyroid status, however all three thyroid autoantibodies positive was seen exclusively in the subclinical hyperthyroid status. Both anti-TPO and thyroglobulin antibody positive titre was relatively higher in subclinical hyperthyroid state however, TSH receptor antibody (TRAb) was seen exclusively in subclinical hyperthyroid state as depicted in Table 3.

**Table 3:** Thyroid Antibody positivity rate in different clinical status

Thyroid Antibody	Clinical Status		p-value
	Subclinical Hyperthyroid	Subclinical Hypothyroid	
Negative	4 (26.7%)	11 (73.3%)	0.007**
One Ab Positive	3 (37.5%)	5 (62.5%)	
Two Abs Positive	10 (33.3%)	20 (66.7%)	
Three Abs Positive	7 (100%)	0	
**Total**	**24**	**36**	

**p value < 0.001 is considered to be statistically significant

The prevalence of anti-TPO and anti-thyroglobulin positive rates in subclinical hypothyroidism were 58.3% and 66.7% respectively (Table 4). Anti-TPO and anti-thyroglobulin was positive mostly in individuals with TSH more than 10 µIU/ml (Table 5). The prevalence of anti-TPO, TRAb and anti-thyroglobulin positive rates in subclinical hyperthyroidism were 75%, 50% and 58.3% as shown in Table 4.

There was a statistically significant relation of TRAb positivity and subclinical hyper- and hypothyroid status.

**Table 4:** Cross-tabulation of thyroid autoantibody positivity and subclinical thyroid status

Thyroid Antibody	Clinical Status				p-value
	Subclinical Hyperthyroid n(%) (N = 24)	Mean ± SD	Subclinical Hypothyroid n(%) (N = 36)	Mean ± SD	
**Anti-TPO**: Positive	18 (75%)	681.02 ± 376.65	21 (58.3%)	565.93 ± 362.08	0.185
**TRAb**: Positive	12 (50%)	15.62 ± 12.34	0		< 0.001
**TGA**: Positive	14 (58.3%)	1547.74 ± 1113.01	24 (66.7%)	1170.27 ± 1042.57	0.512

We found that the autoantibodies positivity in subclinical hypothyroid states in different TSH levels depicted that TSH greater than 10 µIU/ml is present in 15 subclinical hypothyroid patients with TGA positivity and 13 subclinical hypothyroid patients with Anti TPO positivity. This signifies that in our population higher proportion of subclinical hypothyroid patients has TGA positivity as depicted in Table 5.

**Table 5:** Thyroid antibodies in subclinical hypothyroid states in different TSH levels

Range of TSH (µIU/ml)	Anti-TPO		TGA	
	Negative	Positive	Negative	Positive
4.5 to 6.9	2	2	1	3
7.0 to 9.9	4	6	4	6
More than or equal to 10	9	13	7	15

### Correlation between thyroid hormone profile and thyroid autoantibodies

Correlation of thyroid hormone profile and thyroid autoantibodies in subclinical hyperthyroidism revealed that there was a negative correlation between TSH and TRAb. A significant positive correlation was seen between Anti-TPO and TGA (p < 0.001) as well as Anti-TPO and TRAb (p = 0.017) in subclinical hyperthyroid patients as illustrated in Table 6. Similarly in subclinical hypothyroidism, a positive correlation was seen between Anti-TPO and TGA (p < 0.001) while a negative correlation between TGA and TRAb (p = 0.016) as depicted in Table 6 and Table 7.

**Table 6:** Correlation between thyroid hormone profile and thyroid autoantibodies in the subclinical hyperthyroid patients

Variables	Correlation Coefficient (r)	p-value
Gender and TRAb	− 0.602	0.002
FT3 and FT4	0.762	< 0.001
TRAb and TSH	− 0.464	0.022
Anti-TPO and TRAb	0.482	0.017
Anti-TPO and TGA	0.781	< 0.001

**Table 7:** Correlation between thyroid hormone profile and thyroid autoantibodies in the subclinical hypothyroid patients

Variables	Correlation Coefficient (r)	p-value
FT3 and FT4	− 0.385	0.020
TRAb and FT3	− 0.406	0.014
FT4 and TSH	0.704	< 0.001
TGA and TRAb	− 0.397	0.016
Anti-TPO and TGA	0.707	< 0.001

## Discussion

In this hospital-based cross-sectional study in central Nepal, a total of 60 patients were enrolled with subclinical thyroid functions comprising 40% subclinical hyperthyroid and 60% subclinical hypothyroid clinical states. This study highlights the increasing prevalence of thyroid autoimmunity among the study participants as compared to other studies. Most of the study participants were in the reproductive age groups which may perhaps be due to the contingency of fertility issues among the participants that may have accentuated screening for thyroid profiles.

Mostly females presented with subclinical thyroid dysfunctions including both hyper- and hypothyroid status accounting for up to 70.83% (n = 17) and 66.67% (n = 24) respectively. This is in concord with the findings of various studies which claim that females have a higher incidence of thyroid dysfunctions [29–31]. Similar to our studies, Baral et al. [32] and Sharma et al. [20] reported that majority of thyroid dysfunctions occur in active age group i.e. 21–40 years and 15–44 years respectively. In contrary to various other studies which report thyroid dysfunctions predominantly in the older age [33], in our study, there is a paradigm shift of occurrence of thyroid dysfunction in adult age group (below 45 years).

The most common cause of thyroid disorder is iodine deficiency globally while, in iodine-replete areas, autoimmune disease is evident in thyroid disorders [34]. Nepal is a mountainous landlocked country far from sea. It lies in the region called as “Himalayan Goitre Belt” [35, 36]. In pursuit of eradicating iodine deficiency disorder in Nepal, various national level programs were launched including coordinated salt iodization strategies by early 1970s [37]. A remarkable success in elimination of goiter from 55% (1965) to 0.4% (2007) was revealed along with improvement in urinary iodine concentration in school-aged children [38]. This rapid decline, however was deemed deceitful to an extent. A study in Nepal disclosed that a majority of household salt samples had excessive iodine than the WHO recommended values [39]. Excessive iodine intake can be a cause of autoimmune thyroiditis due to a number of reasons including cytokines storm-induced oxidative damage, lipid oxidation, and augmentation of antigenicity of thyroid proteins [40, 41].

In our study, a vast majority of individuals were positive for thyroid antibodies up to 75% and females had higher positive rates. Our finding is in concordance with other studies from Nepal by Shrestha et al. which report significant anti-TPO titre in 26.7% participants [24]. This is also in agreement with the findings of other studies where the occurrence of autoimmune thyroid diseases, both Hashimoto’s thyroiditis, and Grave’s disease is more common in females as compared to males [42].

The present study reports the higher prevalence of anti-TPO antibodies in subclinical hypothyroid compared to subclinical hyperthyroid states. Similar finding is deciphered by Somwaru et al. [43] with higher anti-TPO in subclinical hypothyroid as compared to euthyroid population. However, their prevalence of positive anti-TPO status (35%) is low compared to our study in subclinical hypothyroid status i.e. 58.3%.

Thyroid autoantibody against thyroglobulin in subclinical hypothyroidism (66.7%) marginally superseded subclinical hyperthyroidism (58.3%) in our study. This is very high as compared to those reported in previous studies in eastern Nepal accounting to 25.8% in school children [26]. This value is also comparable to the prevalence in children (14.7%) and that in adults (69.7%) in Sri Lanka [44]. In context of the studies in pregnant population, Kiran et al. reported thyroid peroxidase antibodies and anti-thyroglobulin antibodies positive rates in 66.4% and 52.1% respectively [45]. Similar studies in different cohorts in Nepal by Chaudhary et al. [25] reported anti-TPO positivity in pregnant women to be 3.26% while Tuladhar et al. [46] reported 7.4% anti-TPO positivity in infertile females.

Though the study population in our context include mostly adults, we can observe the gradual shift in the increase in the prevalence of autoimmune thyroid dysfunctions from this study. Despite the shortcoming of our study to access the iodine status in the individuals hand in hand, rise in thyroglobulin antibodies in our context is similar to the scenario in other parts of Nepal and the world owing to the increased consumption of iodine [26, 39, 40]. Overt iodine intake escalating the prevalence of autoimmune thyroid disease is also evident in developed settings like China and Europe(47–48).

TRAb was positive in 50% of the subclinical hyperthyroid population which accounts for 20% (n = 12) of the total participants. There was a significant correlation between TSH and TRAb in subclinical hyperthyroidism in our population which is probably not reported in the Nepalese population as far as we know. We could not prospectively assess the outcome of these individuals with TRAb positive titre. However, the literature suggests that Grave’s disease developed in 12.8% of the euthyroid population with TRAb positive titres [49].

The clinical intervention in the treatment of subclinical hypothyroidism is still debatable [15]. Anti-TPO positivity is associated with subclinical hypothyroidism and hypertension (50–51). It is documented that the presence of thyroid antibodies can predict the progression of subclinical to overt hypothyroidism [52]. Likewise, the presence of thyroid antibodies has been associated with the increased incidence of endothelial dysfunction leading to atherosclerosis [53]. Thyroid autoimmunity is also associated with poor outcomes in pregnancy [54] and hence, American Thyroid Association (ATA) recommends levothyroxine initiation in subclinical hypothyroidism with positive antibody status to avoid poor pregnancy outcomes [55]. Subclinical hypothyroidism may be confused with transient rise in TSH as in advanced age, non-thyroidal illness, analytical interference and renal failure. If untreated, it can predispose to increased cardiovascular risk and cognitive decline [56]. Anti-TPO rises prior to exacerbation of thyroid dysfunction, therefore early screening of these antibodies along with TSH can minimize the potential risk of morbidity and mortality [57, 58]. High anti-TPO antibody positivity status in the reproductive age group in our study could also support the essence of universal thyroid screening for pregnant females and newborns.

The study population taken in our study was small so it may cause bias in coverage of only the population who could visit the tertiary center in Nepal and approximated prevalence based on a single study can be altered. It is advocated to screen all three thyroid antibodies in the larger population.

## Conclusion

Despite subclinical hyper- and hypothyroid status, a flagrant rise in positive titres of thyroid autoantibodies is amenable to the incidence of overt diseases in the long term, which urges timely screening for thyroid autoantibodies in these individuals. Our study has the limitation of not having urine iodine done in the patients to put forward the hypothesis of a causal association between the rising antibody titer and the over-supplementation of iodine in Nepal. It is recommended to screen thyroid autoantibodies in conjunction with iodine status of the study population to scrutinize the real scenario.

## Data Availability

The dataset analyzed during the work is not publicly accessible but can be made available from the corresponding author on reasonable request.

## Abbreviations

anti-TPO: Anti-thyroid peroxidase
AITD: Autoimmune thyroid disorders
ft3: Free triiodothyronine
ft4: Free thyroxine
GD: Grave’s disease
HD: Hashimoto’s disease
TGA: Anti-thyroglobulin
TRAb: Anti-thyroid stimulating hormone receptor
TSH: Thyroid-stimulating hormone
TUTH: Tribhuvan University Teaching Hospital
